# “Very low attenuated plaque on CT appearing as a cyst-like structure in IVUS”

**DOI:** 10.1007/s10554-022-02790-x

**Published:** 2023-01-09

**Authors:** Youssef S. Abdelwahed, Patrick T. Siegrist, Carsten Skurk, Ulf Landmesser

**Affiliations:** 1 Department of Cardiology, Charité – Universitätsmedizin Berlin, Campus Benjamin Franklin, Hindenburgdamm 30, 12203 Berlin, Germany; 2 partner site Berlin, DZHK (German Centre for Cardiovascular Research), Berlin, Germany; 3 HerzZentrum Hirslanden Zurich, Zürich, Switzerland; 4 Berlin Institute of Health (BIH), Berlin, Germany

## Article

**Fig. 1:**
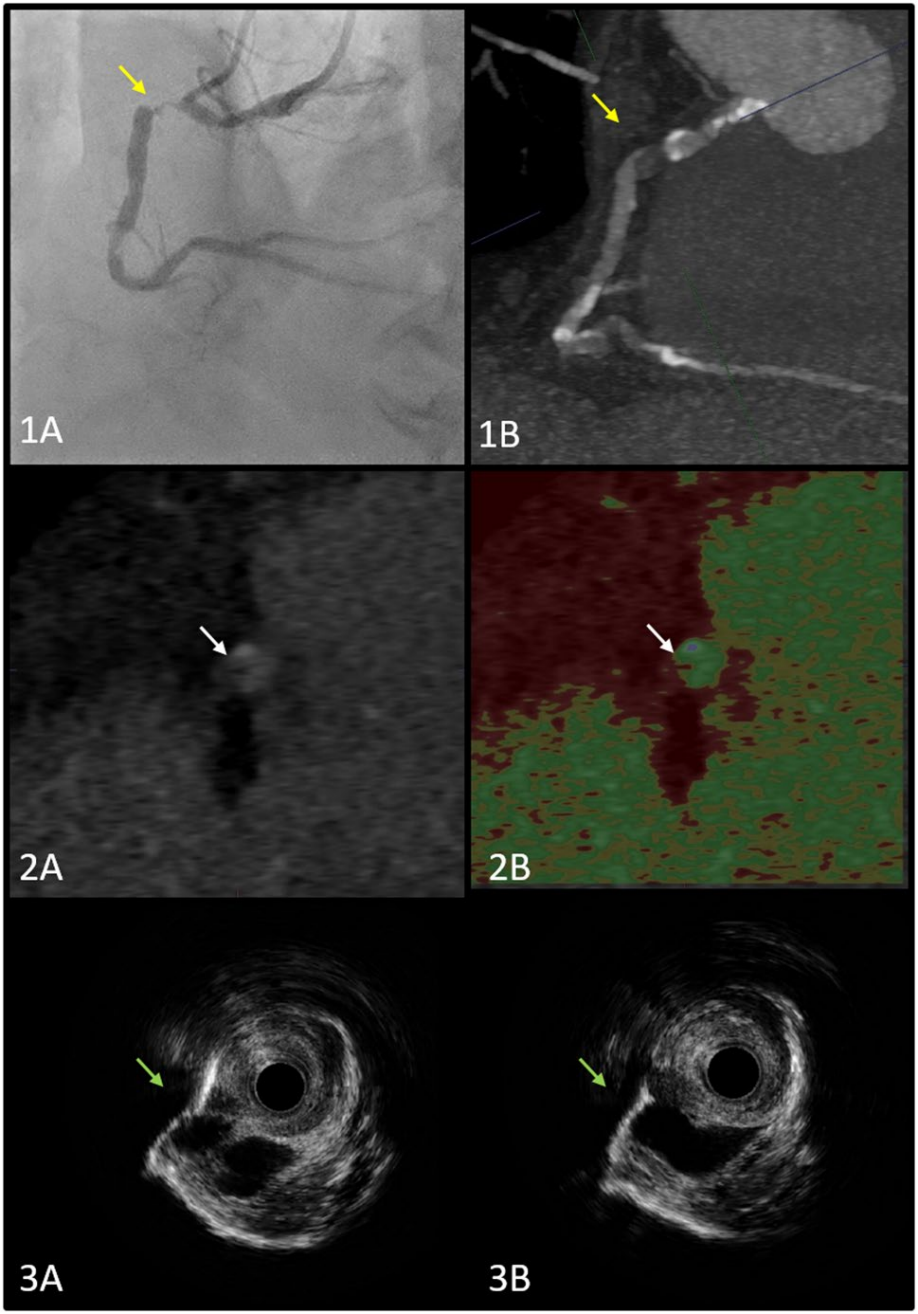
1 **A**–**B** A tubular high grade stenosis can be seen in the proximal part of the RCA and the corresponding CT Image (Yellow Arrows). 2 **A** Cross-sectional view in CT showing the VLAP (White Arrow). 2**B** The VLAP being illustrated using the color mode overlay, with the White arrow pointing at the VLAP. 3 **A**–**B** IVUS imaging showing the Cyst like structure (Green Arrows).

A 72-year-old gentleman with a recurring recurrent chest pain (CCS 2) was electively admitted for a coronary angiography after showing a proximal high grade right coronary artery (RCA) stenosis on a Coronary Computer tomography (CCT) that was performed on outpatient basis. Coronary Angiography confirmed the CCT findings, showing a subtotal stenosis of the proximal RCA (Fig. 1A–B). Detailed analysis of the RCA plaque on cross-sectional CCT images revealed a very low attenuated plaque (VLAP) within the stenosis (Fig. 1C–D). We here thought of performing intracoronary imaging using high definition intravascular ultrasound (HD-IVUS) to better understand the plaque characteristics. HD-IVUS analysis showed a well-defined cyst like structure within the Plaque (Fig. 1E). PCI to the RCA was completed without any complications (Fig. 1E).

Cyst like fluid structures are rarely seen on IVUS. Reports have shown that echolucent and echoattenuated plaques contribute mainly to lipid plaques and necrotic cores. Spotty Calcifications were also reported as a cause. However, well demarcated and defined cyst like structures have been rarely documented in the literature.

